# Depletion Effects Reduce the Preference for Distraction (but Not Reappraisal) During Emotion Regulation

**DOI:** 10.1002/pchj.70021

**Published:** 2025-06-04

**Authors:** Ruiwen Huang, Mei Le, JiaJin Yuan, Jiemin Yang

**Affiliations:** ^1^ Institute of Brain and Psychological Science, Sichuan Normal University Chengdu China; ^2^ Sichuan Key Laboratory of Psychology and Behavior of Discipline Inspection and Supervision Sichuan Normal University Chengdu China

**Keywords:** choice, depletion, emotion regulation, resource

## Abstract

Emotion regulation choice (ERC) requires cognitive control resources. However, it remains unknown whether and how individuals' internal resource levels may influence their ERC. To address this question, 51 participants first performed a depletion or non‐depletion cognitive task. Then, they were presented with negative pictures of high and low intensity and were required to choose distraction or reappraisal, to regulate their emotion, or watch (no regulation) the negative stimulus. The results indicated that individuals showed a greater usage preference for distraction in high‐intensity emotional situations. Besides, individuals' choice of reappraisal was not affected by emotional intensity. More importantly, the depletion effects led to a significant increase in the choice of watch and reduced the choice of distraction (but not reappraisal). These results suggest that cognitive depletion weakens emotion regulation willingness and has different effects on distraction and reappraisal.

## 1 Introduction

Various self‐control can cause ego depletion, which leads to poorer performance in subsequent emotion regulation (Grillon et al. 2015). Successful emotion regulation depends not only on the ability to implement strategies but also on the flexible choice of emotion regulation strategies (Aldao et al. 2010; Gutentag et al. 2016). More importantly, using maladaptive regulatory strategies may result in various forms of psychopathology, such as depression and anxiety (Eftekhari et al. 2009). Therefore, it assumes particular importance to better understand the effects of depletion on the emotion regulation choice (ERC) processes.

ERC is considered to be the cognitive process of making choices between various regulatory strategies in a given context (Sheppes 2014). This dynamic selection involves evaluating strategy effectiveness and resource costs while balancing situational constraints (Blanke et al. 2022; Sheppes 2020). In other words, it implies exerting control over inner emotional reactions by choosing between inner cognitive processes. Increasing evidence suggests the adaptiveness of emotion regulation strategy varies in different contexts, and healthy individuals can fit with differing situational demands by flexibly choosing between regulatory strategies (Aldao et al. 2010).

Cognitive control refers to a series of top‐down mental processes that involve attentional control and regulate automatic responses, which are used to execute goal‐directed behaviors such as making choices (Miyake et al. 2000; Hofmann et al. 2012). Decision‐making is defined as selecting the best option among various available alternatives (Swami 2013). During decision‐making, people weigh the advantages and disadvantages of each option, which involves information processing and cognitive control. These tradeoffs are considered to be the primary reason for resource depletion in decision‐making (Wang et al. 2010). As a special case of decision‐making, ERC is no exception. For example, the engagement and disengagement of emotion regulation strategies create different cost–benefit tradeoffs in the ERC process (Sheppes 2014; Sheppes and Meiran 2008). To be specific, distraction avoids direct cognitive conflict by blocking emotional stimulus before it captures selective attention. Conversely, cognitive reappraisal costs more cognitive resources on emotional information processing, resulting in direct cognitive conflict. This differential resource demand is rooted in their distinct processing stages: distraction operates at the pre‐attentive stage, whereas reappraisal requires higher‐order cognitive elaboration. Recent reviews highlight that such strategic tradeoffs reflect not only cognitive resource allocation but also dynamic shifts in motivational priorities (Forestier et al. 2022), suggesting ERC may involve both capacity limitations and willingness to engage control. Thus, individuals prefer distraction under high‐intensity negative emotional situations. However, in low‐intensity negative emotional situations, individuals mostly prefer reappraisal (Sheppes and Levin 2013). This situational preference reflects a rational cost–benefit analysis where individuals strategically allocate resources based on emotional intensity.

According to the strength model, various acts of cognitive control rely on a common limited resources that can be temporarily depleted (Baumeister and Vohs 2007; Levav et al. 2010; Vohs et al. 2008). However, contemporary critiques argue that this “muscle metaphor” oversimplifies the multicomponent nature of self‐control fatigue, which encompasses resource depletion, motivational shifts, and capacity limitations (Forestier et al. 2022). This concept is analogous to a smartphone battery: even though different apps (cognitive tasks) use different functions, they all draw from the same power source. Notably, converging evidence supports the resource‐sharing hypothesis: cognitive tasks requiring attentional control (e.g., Stroop task) and decision‐making (e.g., product selection) deplete a common resource pool, impairing subsequent performance in unrelated domains (Vohs et al. 2008). For example, resource depletion in cognitive tasks can diminish subsequent cognitive control abilities. Just as lifting weights exhausts physical strength, completing mentally demanding tasks reduces available resources for future challenges. Previous studies have shown that making choices results in impairment in subsequent cognitive tasks involving attentional control (Vohs et al. 2008). Imagine shopping for hours, carefully comparing products: by the end, you might struggle to focus on reading a book. Conversely, it is also true that a prior attentional control task would undermine subsequent choice behavior (Baumeister et al. 1998). Meanwhile, previous studies have found that individual differences in cognitive resources can partly predict their choice of regulatory strategies. Specifically, children with lower executive resources (e.g., working memory capacity) have preferences for less effortful regulatory strategy (Dorman Ilan et al. 2019). Another study also found that old people preferred strategies with lower cognitive costs for the limited cognitive resources (Scheibe et al. 2015). Critically, recent organizational psychology studies demonstrate that self‐control failures stem from the interplay of diminished resources and altered cost–benefit calculations (Wehrt et al. 2022), highlighting the need to examine how these mechanisms translate to emotion regulation contexts. However, to date, there is no direct evidence of whether an individual's internal resources level influences ERC. This gap is particularly salient in light of the differential resource demands of reappraisal and distraction. Thus, understanding how resource depletion influences ERC has critical implications for interventions targeting emotion dysregulation in clinical populations.

To address this question, the present study manipulated the cognitive resources directly rather than utilizing individual differences to test the impact of depletion effects on ERC. Specifically, we used the classic word/color Stroop task as a resource depletion task, which is frequently used for depletion research, requiring significant effort to complete (Hagger et al. 2010; Koppel et al. 2019; Dang et al. 2021; Lin et al. 2020; Singh and Göritz 2018). This approach aligns with methodological recommendations for inducing genuine self‐control demands through validated cognitive conflict tasks (Forestier et al. 2022). Then we employed a modified ERC paradigm (Sheppes et al. 2014; Sheppes et al. 2011) to measure the strategies preference of participants in various negative emotional intensity contexts. Given individuals' choice of an emotion regulation strategy in real‐life emotional situations is independent of one another, we added the “watch” option to the original forced‐choice paradigm. Accordingly, participants can choose distraction or reappraisal to regulate their emotions, or they can choose to watch (no regulate) the negative stimulus. Thus, the first aim of the current study was to explore the pattern of ERC under the non‐depletion session after adding the watch option. Secondly, we explore the effect of depletion on ERC.

Based on the previous studies on the relationship between resources and ERC, individuals with lower resources tend to use less effortful strategies. Building on evidence that self‐control fatigue involves prioritized conservation of attentional resources, we predict individuals would choose to watch more than regulatory strategies in the depletion session, in that implementing regulatory strategies requires more cognitive effort, and resource depletion in cognitive tasks diminishes this ability. Additionally, previous research has found that attentional control is susceptible to ego depletion. Distraction requires attentional control to turn attention to something irrelevant, whereas reappraisal relies more on semantic processing to reinterpret emotional information. Consistent with the multicomponent model (Forestier et al. 2022), depletion may disproportionately impair strategies requiring sustained attention (e.g., distraction) compared to those involving cognitive reframing. Therefore, we predict that individuals would use less distraction in the depletion session than in the non‐depletion session, in that depletion reduces attentional control.

## 2 Materials and Methods

### 2.1 Participants

Sample size was determined using G*Power 3.1 (Faul et al. 2007) to achieve 80% power (*α* = 0.05, two‐tailed) for detecting a moderate interaction effect (partial *η* ^2^ = 0.25) in a repeated‐measures ANOVA. this calculation yielded a minimum required sample size of 24 participants. to account for potential attrition and data exclusion criteria (e.g., technical errors, insufficient accuracy in practice trials), we recruited 51 participants, ensuring robust statistical power while aligning with prior ERC studies (Sheppes et al. 2014). All experimental procedures were approved by Sichuan Normal University Review Board Ethics Committee. The written informed consent was obtained from the participants, and all procedures were performed in accordance with relevant guidelines and regulations.

### 2.2 Stimuli

One hundred and twenty‐four pictures were selected from the International Affective Picture System (IAPS; Lang et al. 2008), which include 62 high‐intensity negative pictures (mean arousal = 6.38; mean valence = 2.61), 62 low‐intensity pictures (mean arousal = 4.88; mean valence = 3.17), *p* < 0.001. Previous studies have confirmed that the arousal and valence difference between low‐intensity and high‐intensity stimuli is sufficient to induce different levels of emotional response activation (Bradley et al. 2001).

### 2.3 Procedure

**FIGURE 1:**
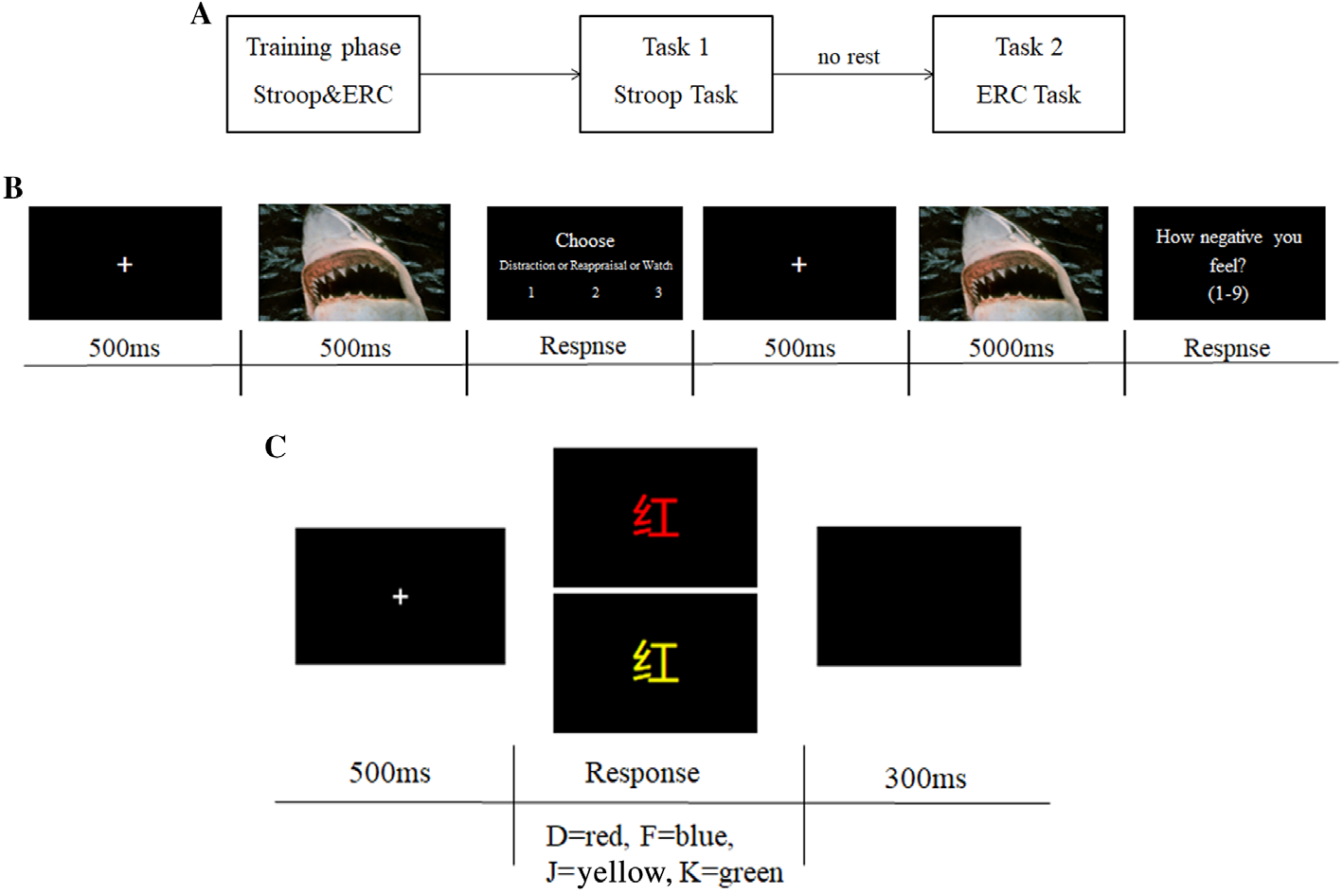
(A) The organization of behavioral procedure of the study. (B) The schematic illustration of the emotion‐regulation choice task in task 2. (C) The schematic illustration of the resource depletion task in task 1.

The present study consisted of two experimental sessions, and the procedure can be found in Figure 1A. For half the subjects, the depletion and non‐depletion sessions took place on the first and second visits, respectively. For the other half of the subjects, this order was reversed. The two sessions were similar except for the Stroop tasks. Subjects performed the Stroop task first, and the ERC task started immediately to ensure the depletion effect.

#### 2.3.1 Questionnaire Measurement

After the participants arrived at the laboratory, they completed the Positive and Negative Affect Schedule (Watson et al. 1988) and the State‐Trait Anxiety Inventory (STAI‐Y; Spielberger 2010) was administered immediately before each experimental session to capture state‐level affective responses prior to resource manipulation. Participants completed the Beck Depression Inventory (BDI‐I3; Smarr and Keefer 2011) during their first laboratory visit to screen for severe depressive symptoms. based on established clinical cutoffs (total score ≥ 16), individuals meeting criteria for severe depression were excluded to control for potential confounding from acute depressive symptoms in ERC.

#### 2.3.2 Training Task

During the training phase, participants were presented with negative pictures and were instructed to think about something unrelated to the picture that was emotionally neutral (distraction), or reinterpret each picture in a constructive way that would keep observers calm (reappraisal), or just watch the pictures carefully and let emotions reveal naturally without suppressing or changing them. To ensure that participants correctly understood regulation strategies, we asked them to explain how they implemented regulatory strategies during the training trials and corrected them when necessary. Each strategy was used two times (one low‐intensity trial and one high‐intensity trial) in the training phase.

#### 2.3.3 Resource Depletion Task

Participants first performed a modified word/color Stroop task, with Chinese color words (“红” = red, “蓝” = blue, “黄” = yellow, “绿” = green) printed in one of these four colors on a black background (see Figure 1C). When the font color matches the font meaning, it is considered a consistent trial, and the reverse is an inconsistent trial (Gailliot et al. 2007). Each trial started with a 500 ms fixation, followed by a color word (“红”, “蓝”, “黄”, “绿”) presented in conflicting/consistent colors, requiring participants to respond based on the displayed font color using assigned keys (“D” = red, “F” = blue, “J” = yellow, “K” = green). Stimuli remained visible until response or 1500 ms timeout, ended by a 300 ms black screen. There were 400 trials, of which the depletion session consisted of 75% inconsistent trials and 25% consistent trials, and the non‐depletion session consisted of 25% inconsistent trials and 75% consistent trials. The formal experiment will only begin after achieving an 85% accuracy rate in practice.

#### 2.3.4 Emotion Regulation Task

The choice phase (see Figure 1B) consists of 114 emotional pictures. Each trial started with a 500 ms fixation, and then an emotional picture was displayed for 500 ms. After that, participants saw the words distraction, reappraisal, and watch presented on the screen, among which they chose by pressing one of three buttons (“1”, “2”, or “3”). The participants then implemented their chosen option during a second presentation of the picture (5000 ms). Finally, participants rated how negative the picture made them feel on a scale of 1 (not negative at all) to 9 (very negative).

#### 2.3.5 Self‐Reports

At the end of all the tasks, participants were asked how difficult and fatigued the Stroop task was on a scale of 1 (*not at all*) to 7 (*very much*).

## 3 Results

Statistical analysis was performed using SPSS Statistics 22.0. The significance level was at 0.05. The Bonferroni–Holm method was used for post hoc comparisons if significant main or interaction effects appeared.

### 3.1 Self‐Reports and Manipulation Check

No significant differences in positive and negative mood or scores on the state anxiety inventory (SAI) were observed between two sessions (Table 1).

**TABLE 1:** Descriptive statistics for PANAS and SAI in the depletion and non‐depletion sessions.

	Non‐depletion (*n* = 51)	Depletion (*n* = 51)	*t*	*p*
SAI (*M* ± SD)	43.31 ± 5.07	41.90 ± 5.65	1.78	0.08
PANAS‐P (*M* ± SD)	2.66 ± 0.93	2.60 ± 0.97	0.6	0.55
PANAS‐N (*M* ± SD)	1.44 ± 0.63	1.39 ± 0.60	0.98	0.33

Abbreviations: PANAS‐N, positive and negative affect schedule‐negative PANAS‐P, positive and negative affect schedule‐positive; SAI, state anxiety inventory.

The react time (RT) of the depletion task (*M* = 650.37, SD = 71.95) is significantly longer than the non‐depletion task (*M* = 702.79, SD = 98.30), while the accuracy (ACC) of the depletion task (*M* = 0.94, SD = 0.04) is significantly lower than that of the non‐depletion task (*M* = 0.95, SD = 0.03). The depletion tasks (*M* = 4.37, SE = 0.13) were rated as more difficult than the control task (*M* = 2.37, SE = 0.11), *t* (50) = −12.62, *p* < 0.0001. The depletion tasks (*M* = 4.29, SE = 0.12) were leading to greater exhaustion than the control task (*M* = 2.04, SE = 0.11), *t* (50) = −15.26, *p* < 0.0001. Thus, the resource depletion manipulation was successful (Table 2).

**TABLE 2:** RT and ACC in the depletion and non‐depletion sessions in Stroop task.

	Non‐depletion (*n* = 51)	Depletion (*n* = 51)	*t*	*p*
ACC (*M* ± SD)	0.95 ± 0.027	0.94 ± 0.039	−4.24	< 0.01
RT (*M* ± SD)	654.00 ± 71.95	702.79 ± 98.30	3.251	< 0.01

Abbreviations: ACC, accuracy; RT, react time.

### 3.2 Emotion Regulation Choice

**FIGURE 2:**
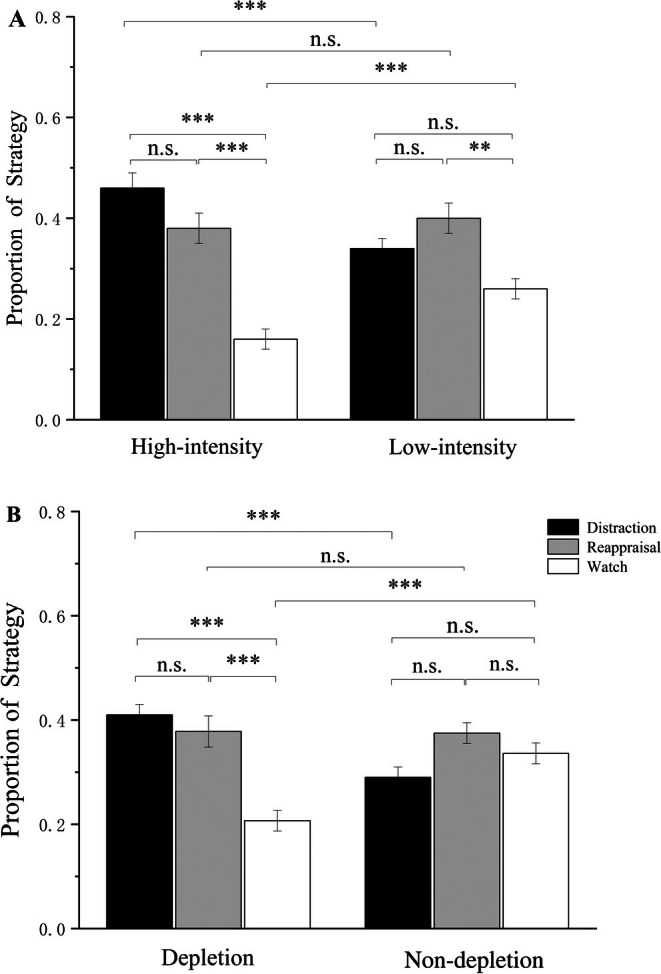
ANOVA of the proportion of strategy. (A) The emotion regulation choice of different emotional intensities in non‐depletion sessions. (B) The emotion regulation choice in different depletion sessions. ***p < 0.001, **p < 0.01, *p < 0.05. Error bars represent SEM.

Firstly, we conducted a 2 (intensity: high, low) × 3 (strategy: distraction, reappraisal, watch) repeated measures ANOVA for the non‐depletion session only. Results revealed a significant main effect of strategy, (*F*(2, 49) = 14.758, *p* < 0.001, $\eta_{p}^{2}$ = 0.228). The main effect of intensity was not significant, (*F*(1, 50) < 0.001, *p* = 0.999, $\eta_{p}^{2}$ < 0.001). Importantly, there was a significant interaction effect of intensity × strategy, (*F*(2, 49) = 20.309, *p* < 0.001, $\eta_{p}^{2}$ = 0.289). The results showed that participants use distraction (*M* = 0.46, SE = 0.03, 95% CI [0.40, 0.51]) and reappraisal (*M* = 0.38, SE = 0.03, 95% CI [0.33, 0.43]) more frequently than watch (*M* = 0.16, SE = 0.02, 95% CI [0.12, 0.20]) in high‐intensity situation. In low‐intensity situation, participants use reappraisal (*M* = 0.40, SE = 0.03, 95% CI [0.35, 0.45]) more frequently than watch (*M* = 0.26, SE = 0.02, 95% CI [0.22, 0.31]), but there was no significant difference between distraction (*M* = 0.34, SE = 0.02, 95% CI [0.29, 0.39]) and watch (see Figure 2A). Moreover, there was no significant difference between distraction and reappraisal in high‐intensity situation or in low‐intensity situation.

Secondly, we conducted a 2 (intensity: high, low) × 2 (session: depletion, non‐depletion) × 3 (strategy: distraction, reappraisal, watch) repeated measures ANOVA to test whether the depletion and non‐depletion sessions would show different strategy choice patterns in the contexts of varied emotional intensities. Results revealed a significant main effect of strategy, (*F*(1, 50) = 5.570, *p* = 0.005, $\eta_{p}^{2}$ = 0.100) and intensity, (*F*(1, 50) = 7.963, *p* = 0.007, $\eta_{p}^{2}$ = 0.137). The main effect of the session was not significant, (*F*(1, 50) = 2.295, *p* = 0.136, $\eta_{p}^{2}$ = 0.044). In addition, the intensity × strategy interaction, (*F*(2, 49) = 29.720, *p* < 0.001, $\eta_{p}^{2}$ = 0.373), the session × strategy interaction, (*F*(2, 49) = 16.695, *p* < 0.001, $\eta_{p}^{2}$ = 0.250), and the three‐way interaction among session, strategy and intensity, (*F*(2, 49) = 5.585, *p* = 0.005, $\eta_{p}^{2}$ = 0.100) were significant. Therefore, we performed the simple effect tests and found that in both sessions, participants chose distraction more in high‐intensity than low‐intensity, and chose watch more in low‐intensity than in high‐intensity. Moreover, in both sessions, there was no significant difference in reappraisal between high‐intensity and low‐intensity. However, in the depletion session, the increase of participants' choice of watch in low‐intensity was larger than in the non‐depletion. More importantly, participants chose watch more frequently in the depletion session (*M* = 0.33, SE = 0.02, 95% CI [0.29, 0.38]) than in the non‐depletion (*M* = 0.21, SE = 0.02, 95% CI [0.17, 0.25]), and chose distraction more frequently in the non‐depletion session (*M* = 0.40, SE = 0.02, 95% CI [0.35, 0.45]) than in the depletion (*M* = 0.30, SE = 0.02, 95% CI [0.27, 0.34]). However, there was no significant difference in reappraisal between sessions (see Figure 2B). These results illustrated that cognitive resource depletion can decrease the choice of emotion regulatory strategies.

## 4 Discussion

In the current study, we manipulated individual's internal cognitive resources directly by using the Stroop task, and compared individual's choice of “distraction”, “reappraisal” and “watch” under different resource depletion situations by a modified ERC paradigm. The results demonstrated that cognitive depletion weakens emotion regulation willingness, and has different effects on distraction and reappraisal.

Previous study found that lower resources level is associated with the preference for less effortful coping approach (Scheibe et al. 2015; Dorman Ilan et al. 2019). From a motivational perspective, the cognitive energetics theory (CET) may help explain the different ERC patterns between depletion and non‐depletion sessions. The CET accounts for the likelihood of purposeful cognitive activity depending on the interaction between driving and restraining forces (Kruglanski et al. 2012). When it comes to ERC, the driving forces involve the motivation to down‐regulate emotion and the availability of cognitive resources. Critically, emerging evidence highlights that implicit emotion recognition mechanisms, such as EEG‐based temporal stability reflecting neural adaptation to emotional intensity, may amplify these driving forces by sharpening sensitivity to high‐intensity stimuli (Ju et al. 2024). The restraining forces involve the task demands, competing goals, and the tendency to conserve resources. Specifically, the intensity of negative emotional stimuli simultaneously affects the driving forces (the motivation to down‐regulate emotion) and restraining forces (the difficulty of implementing strategies) of emotional regulation. In the present study, the driving force was the same in both depletion and non‐depletion sessions, while the depletion task increases the restraining forces (the tendency to conserve resources). With the decrease in available cognitive resources, individuals' inclination to conserve resources increases, resulting in fewer regulatory strategy choices.

Importantly, we found that the depletion effects reduce the use of distraction (but not reappraisal) significantly. There are two tentative explanations for this finding. Firstly, Stroop task requires participants to focus their attention on both the color and meaning of words, suggesting that incongruent trial have higher attentional requirements than congruent trails (Badzakova‐Trajkov et al. 2009). Previous studies have found that ego depletion reduces attention control by disrupting the executive function processes required for sustaining attention under regulatory demands (Englert et al. 2015; Garrison et al. 2018). Whereas distraction recruits greater activation in the right prefrontal and parietal regions associated with attentional control compared to reappraisal (McRae et al. 2010; Sheppes et al. 2011), it is clear that the depletion task reduces the attentional control resources (Garrison et al. 2018), resulting in larger restraining forces on implementing distraction. Secondly, previous studies have shown that reappraisal affordance is an important cognitive factor that may influence ERC (Suri et al. 2018; Young and Suri 2020). Reappraisal affordance refers to the opportunities for altering the meaning of a stimulus from another perspective that is inherent in that stimulus. For instance, a picture of animals or people injured can be easily interpreted in various ways, whereas a picture of dead animals or people is difficult to find a suitable perspective to reinterpret. Reappraisal affordance proved strongly predictive of ERC, where greater reappraisal affordances are associated with higher frequency of reappraisal choices. Thus, we reasoned that participants only choose reappraisal in situations with high reappraisal affordance (low reappraisal generation difficulty), which may require less cognitive effort and therefore be less affected by resource depletion. Importantly, a previous study found that reappraisal affordance has stability across participants and within participants, and was unlikely to be driven by internal, person‐specific variables. These may be possible explanations for resources depletion that did not affect the frequency of reappraisal choices.

In a forced‐choice paradigm individuals chose more reappraisal in low‐intensity situations but more distraction in high‐intensity situations (Sheppes et al. 2014, 2011). Notably, implicit emotion recognition processes, such as EEG temporal stability features (Ju et al. 2024), may calibrate the detection of emotional intensity, which could shift strategy prioritization when a non‐regulatory option (e.g., watch) is introduced. However, we found no difference in participants' choice of distraction and reappraisal after the addition of watch option, whether in high‐ or low‐emotional‐intensity situations. These findings in the current study may provide new evidence for the impact factors on reappraisal that emotional intensity may not always be a determinant of reappraisal. The observed depletion‐driven reduction in regulatory willingness (e.g., increased watch choices) may further inform clinical research on emotion regulation deficits in depression. Notably, depression is associated with chronic cognitive resource depletion and rigid strategy selection (Joormann and Stanton 2016), and recent studies highlight sex‐specific neural markers in this population. For instance, females with depression exhibit distinct cortical structural alterations (e.g., reduced prefrontal thickness; Mou et al. 2023), while sex‐specific neurofunctional patterns differentiate depression severity levels (Li et al. 2024). Such neural markers, combined with behavioral depletion paradigms (e.g., Stroop tasks), could help identify individuals prone to regulatory inflexibility. Future research integrating EEG‐based temporal stability features with clinical assessments may refine diagnostic tools for depression‐related emotion dysregulation (Ju et al. 2024). In a forced‐choice paradigm, the either‐or choice between distraction and reappraisal determined that more choice of distraction implies less choice of reappraisal. However, the addition of the watch option (no regulation) allowed participants to choose the regulatory strategy only when it was appropriate to use distraction or reappraisal. Suri et al. (2018) found that the relation between intensity and ERC may be affected by the inclusion of reappraisal affordances. Specifically, intensity was not related to ERC when reappraisal affordances were considered using vignettes, and intensity was a significant predictor of ERC when omitted reappraisal affordances from the model. Additionally, we observed more watch choices in low‐intensity situations than in high‐intensity situations, which can also be explained by CET that high‐intensity negative stimuli are associated with higher driving forces (the motivation to down‐regulate emotion).

Several limitations of this study should be discussed. First, although our resource depletion manipulation was effective, the Stroop task mainly involves inhibitory control. There are three types of cognitive control: inhibition control, interference control, and cognitive flexibility. Different tasks require the involvement of various kinds of control processes (Diamond 2013). Further studies should therefore consider exploring the impact of different types of cognitive resource depletion on ERC. Second, distraction and reappraisal have been found to be associated with various cognitive costs in previous studies. Noticeably, reappraisal includes multiple sub‐strategies which may involve different cognitive costs. Future studies should examine the impact of depletion on sub‐strategies during the ERC process. Furthermore, distraction and reappraisal draw upon different neural networks that could be differently affected by resource depletion. Future studies should incorporate neurophysiological methods to further investigate the impact of depletion on two strategies.

To conclude, this study found that the tendency of individuals to regulate emotions is influenced by cognitive resources. Specifically, the cognitive depletion weakened emotion regulation willingness. The results directly demonstrated that ERC depends not only on individual differences in executive capacity but also on the extent to which these resources are being exhausted within the individuals. Healthy physical and mental functioning requires regulating inappropriate emotional responses in daily life with adaptive approaches. These control mechanisms require cognitive resources and may also be impaired by depletion. Future studies will be needed to focus on finding factors that protect against the negative impact of depletion on ERC.

## Ethics Statement

This study was approved by the ethical committee of human research at Sichuan Normal University. We declare this approval in the manuscript (Method‐Participants).

## Conflicts of Interest

The authors declare no conflicts of interest.

## Data Availability

The data that support the findings of this study are available on request from the corresponding author, Jiemin Yang, upon reasonable request.
